# Supplementary material for: Avirulins, a Novel Class of HIV-1 Reverse Transcriptase Inhibitors Effective in the Female Reproductive Tract Mucosa
Source: Viruses. 2019 May 1;11(5):408. doi: 10.3390/v11050408 (PMC6563246; doi:10.3390/v11050408)
Supplement: Supplementary file 1 [file viruses-11-00408-s001.pdf]

viruses

Supplementary Table S1. Avirulin chemical properties

	IC50 (µM)	Molecular Weight	IUPAC
High Activity	**1.5- 3.5**		
Av-5	1.57 ±0.42	433.43	3-[1-(1-methyl-1H-pyrazole-4-carbonyl)pyrrolidin-3-yl]-5-{2-[2-(trifluoromethyl)phenoxy]ethyl}-1H-pyrazole
Av-26	2.17 ±0.30	397.39	2-methoxy-1-[3-(5-{2-[2-(trifluoromethyl)phenoxy]ethyl}-1H-pyrazol-3-yl)pyrrolidin-1-yl]ethan-1-one
Av-27	2.30 ±0.61	435.48	3-(1-cyclohexanecarbonylpyrrolidin-3-yl)-5-{2-[3-(trifluoromethyl)phenoxy]ethyl}-1H-pyrazole
Av-14	3.59 ±0.45	399.87	5-[2-(2-chlorophenoxy)ethyl]-3-[1-(1-methyl-1H-pyrazole-4-carbonyl)pyrrolidin-3-yl]-1H-pyrazole
Moderate Activity	**3.6-15**		
Av-11	3.61 ±1.11	447.45	3-[1-(1-ethyl-1H-pyrazole-3-carbonyl)pyrrolidin-3-yl]-5-{2-[3-(trifluoromethyl)phenoxy]ethyl}-1H-pyrazole
Av-28	5.43 ±2.20	448.44	2,4-dimethyl-5-[3-(5-{2-[2-(trifluoromethyl)phenoxy]ethyl}-1H-pyrazol-3-yl)pyrrolidine-1-carbonyl]-1,3-oxazole
Av-29	11.32 ±4.82	399.87	5-[2-(2-chlorophenoxy)ethyl]-3-[1-(3-methyl-1H-pyrazole-4-carbonyl)pyrrolidin-3-yl]-1H-pyrazole
Av-8	12.65 ±4.86	447.45	3-[1-(1-ethyl-1H-pyrazole-3-carbonyl)pyrrolidin-3-yl]-5-{2-[4-(trifluoromethyl)phenoxy]ethyl}-1H-pyrazole
Low Activity	**25-100 µM**		
Av-22	27.28 ±11.6	447.5	1-ethyl-3-methyl-4-{[3-(5-{2-[3-(trifluoromethyl)phenoxy]ethyl}-1H-pyrazol-3-yl)pyrrolidin-1-yl]methyl}-1H-pyrazole
Av-7	60.25 ±2.05	433.47	3-{1-[(1-ethyl-1H-pyrazol-4-yl)methyl]pyrrolidin-3-yl}-5-{2-[4-(trifluoromethyl)phenoxy]ethyl}-1H-pyrazole
Av-13	44.17 ±38.37	433.47	1,3-dimethyl-4-{[3-(5-{2-[3-(trifluoromethyl)phenoxy]ethyl}-1H-pyrazol-3-yl)pyrrolidin-1-yl]methyl}-1H-pyrazole
Av-2	~50	433.43	3-[1-(1-methyl-1H-pyrazole-4-carbonyl)pyrrolidin-3-yl]-5-{2-[3-(trifluoromethyl)phenoxy]ethyl}-1H-pyrazole
Av-4	>50	433.43	3-[1-(3-methyl-1H-pyrazole-4-carbonyl)pyrrolidin-3-yl]-5-{2-[4-(trifluoromethyl)phenoxy]ethyl}-1H-pyrazole
Av-6	>50	419.44	3-{1-[(1-methyl-1H-pyrazol-4-yl)methyl]pyrrolidin-3-yl}-5-{2-[4-(trifluoromethyl)phenoxy]ethyl}-1H-pyrazole
Av-10	>50	383.42	5-[2-(3-fluorophenoxy)ethyl]-3-[1-(1-methyl-1H-pyrazole-4-carbonyl)pyrrolidin-3-yl]-1H-pyrazole
Av-12	>50	433.47	1,3-dimethyl-4-{[3-(5-{2-[4-(trifluoromethyl)phenoxy]ethyl}-1H-pyrazol-3-yl)pyrrolidin-1-yl]methyl}-1H-pyrazole
Av-16	>50	419.4	3-[1-(1H-pyrazole-3-carbonyl)pyrrolidin-3-yl]-5-{2-[3-(trifluoromethyl)phenoxy]ethyl}-1H-pyrazole
Av-20	>50	433.43	3-[1-(1-methyl-1H-pyrazole-5-carbonyl)pyrrolidin-3-yl]-5-{2-[3-(trifluoromethyl)phenoxy]ethyl}-1H-pyrazole
Av-21	>50	399.87	5-[2-(3-chlorophenoxy)ethyl]-3-[1-(1-methyl-1H-pyrazole-4-carbonyl)pyrrolidin-3-yl]-1H-pyrazole

Av-24	>50	421.46	3-(1-cyclopentanecarbonylpyrrolidin-3-yl)-5-{2-[4-(trifluoromethyl)phenoxy]ethyl}-1H-pyrazole
Av-25	>50	430.42	3-[3-(5-{2-[3-(trifluoromethyl)phenoxy]ethyl}-1H-pyrazol-3-yl)pyrrolidine-1-carbonyl]pyridine
Av-30	>50	399.87	5-[2-(3-chlorophenoxy)ethyl]-3-[1-(3-methyl-1H-pyrazole-4-carbonyl)pyrrolidin-3-yl]-1H-pyrazole
Av-31	>50	379.42	3-[1-(cyclopropylmethyl)pyrrolidin-3-yl]-5-{2-[2-(trifluoromethyl)phenoxy]ethyl}-1H-pyrazole
Av-18	>50	447.45	3-(1H-pyrazol-1-yl)-1-[3-(5-{2-[3-(trifluoromethyl)phenoxy]ethyl}-1H-pyrazol-3-yl)pyrrolidin-1-yl]propan-1-one
No Activity			
Av-9	>100	447.5	1-ethyl-3-methyl-4-{[3-(5-{2-[4-(trifluoromethyl)phenoxy]ethyl}-1H-pyrazol-3-yl)pyrrolidin-1-yl]methyl}-1H-pyrazole
Av-19	>100	395.46	5-[2-(2-methoxyphenoxy)ethyl]-3-[1-(1-methyl-1H-pyrazole-4-carbonyl)pyrrolidin-3-yl]-1H-pyrazole
Av-23	>100	395.46	5-[2-(4-methoxyphenoxy)ethyl]-3-[1-(1-methyl-1H-pyrazole-4-carbonyl)pyrrolidin-3-yl]-1H-pyrazole
Av-15	>100	407.51	3-[1-(1-methyl-1H-pyrazole-4-carbonyl)pyrrolidin-3-yl]-5-{2-[4-(propan-2-yl)phenoxy]ethyl}-1H-pyrazole
Av-1	>100	433.43	3-[1-(1-methyl-1H-pyrazole-4-carbonyl)pyrrolidin-3-yl]-5-{2-[4-(trifluoromethyl)phenoxy]ethyl}-1H-pyrazole
Av-32	>100	433.43	3-[1-(3-methyl-1H-pyrazole-4-carbonyl)pyrrolidin-3-yl]-5-{2-[3-(trifluoromethyl)phenoxy]ethyl}-1H-pyrazole
Av-3	>100	447.45	1,3-dimethyl-4-[3-(5-{2-[4-(trifluoromethyl)phenoxy]ethyl}-1H-pyrazol-3-yl)pyrrolidine-1-carbonyl]-1H-pyrazole
Av-17	>100	433.47	3-{1-[(1,5-dimethyl-1H-pyrazol-4-yl)methyl]pyrrolidin-3-yl}-5-{2-[4-(trifluoromethyl)phenoxy]ethyl}-1H-pyrazole

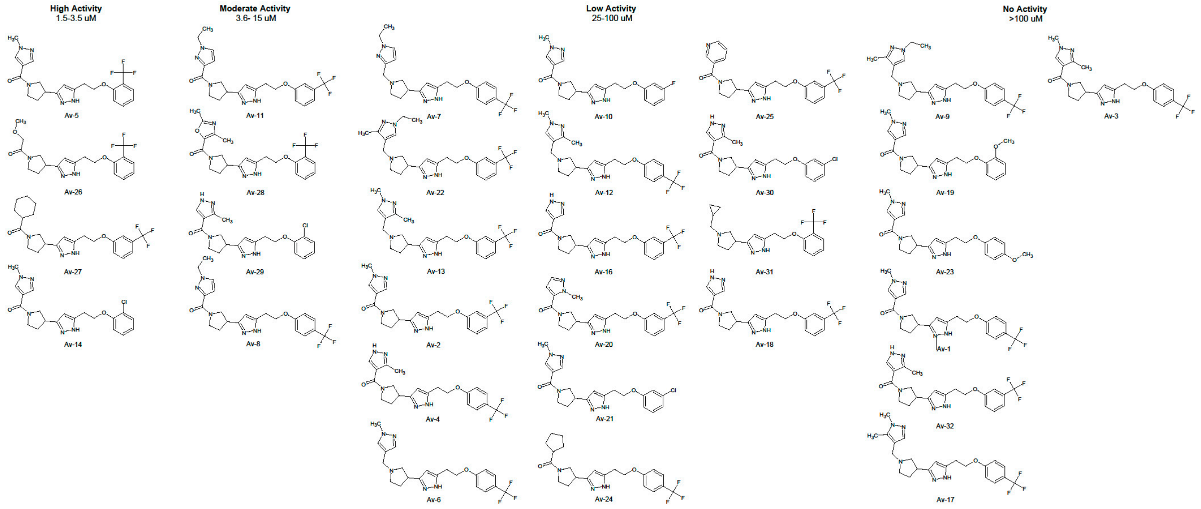

(**a**)

Supplementary Figure S1. Structure of Avirulins.
